# Cross-modal transfer in visual and nonvisual cues in bumblebees

**DOI:** 10.1007/s00359-019-01320-w

**Published:** 2019-03-11

**Authors:** Michael J. M. Harrap, David A. Lawson, Heather M. Whitney, Sean A. Rands

**Affiliations:** School of Biological Sciences, University of Bristol, Life Sciences Building, Tyndall Avenue, Bristol, BS8 1TQ UK

**Keywords:** Cross-modality, Floral temperature, Pattern learning, Spatial patterns, Bumblebees

## Abstract

Bumblebees *Bombus terrestris* are good at learning to distinguish between patterned flowers. They can differentiate between flowers that differ only in their patterning of scent, surface texture, temperature, or electrostatic charge, in addition to visual patterns. As recently shown, bumblebees trained to discriminate between nonvisual scent patterns can transfer this learning to visually patterned flowers that show similar spatial patterning to the learnt scent patterns. Bumblebees can, therefore, transfer learnt patterns between different sensory modalities, without needing to relearn them. We used differential conditioning techniques to explore whether cross-modal transfer of learnt patterns also occurred between visual and temperature patterns. Bumblebees that successfully learnt to distinguish rewarding and unrewarding temperature patterns did not show any preferences for the corresponding unlearnt visual pattern. Similarly, bumblebees that learnt visual patterns did not transfer these to temperature patterns, suggesting that they are unable to transfer learning of temperature and visual patterns. We discuss how cross-modality pattern learning may be limited to modalities that have potentially strong neurological links, such as the previously demonstrated transfer between scent and visual patterns.

**Electronic supplementary material:**

The online version of this article (10.1007/s00359-019-01320-w) contains supplementary material, which is available to authorized users.

## Introduction

Many floral displays show structured or patterned signals that can be learned by pollinators, and these patterns can show differences in the intensity, composition, and location of their components across the flower (Hempel de Ibarra et al. 2015). Visual patterns are the best understood example, where the patterns include differences in colouring and brightness across the flower. In bee-pollinated flowers, this most frequently involves a darker coloured flower centre (in terms of the green sensitive L-receptor found in bees), and a brighter periphery (Hempel de Ibarra and Vorobyev 2009), and these patterns are more salient than unpatterned flowers to insect pollinators (Johnson and Dafni 1998; Spaethe et al. 2001; Hempel de Ibarra et al. 2001). Flowers can also create other learnable visual patterns such as linear floral guides (Lawson and Rands 2018), polarization patterns (Foster et al. 2014), or iridescence (Whitney et al. 2009b; but see; Kjernsmo et al. 2018). Nonvisual patterns are also common, and include scent patterns (where different amounts of floral volatiles or different floral volatile chemicals are released across the flower; Bergström et al. 1995; Balao et al. 2011; Lawson et al. 2018), electrostatic patterns (where properties of the flower allow charge to accumulate differentially across the flower surface and between flowers: Clarke et al. 2013), texture patterns (where shape of cells on the flower surface differ: Kevan and Lane 1985), and temperature patterns (where different parts of the flower differ in how they heat up: Harrap et al. 2017). All these pattern types have been demonstrated to differ between flower species and can be used by pollinators for learning flower identity.

While there is plenty of research exploring the signal functions of singular modalities, there is still much to learn about why flowers display multiple modalities simultaneously (Raguso 2004), especially when considering the potential costs of multimodal displays such as the risk of attracting herbivores or the metabolic costs of complex display components (Helsper et al. 1998; Theis 2006; Kaczorowski et al. 2012). This aspect of pollination biology has been largely underappreciated in terms of pollinator behaviour, with Leonard et al. (2011) noting that over the preceding 2 decades only 5% of journal articles on bee learning clearly considered bees’ responses to multimodal stimuli. However, in recent years, it seems that there has been a resurgence in research relating to the nature of multimodal signals (Katzenberger et al. 2013; Riffell and Alarcón 2013; Leonard and Masek 2014).

Studies of multimodality have mostly focussed on the question of why these multimodal signals have evolved and the behavioural response of animals to them (Rowe 1999; Partan and Marler 1999; Thompson et al. 2008). The production and perception of signals in multiple sensory channels also influence the evolution of sensory and perceptual physiology, in addition to speciation and survival, making multimodality significant to multiple fields of research (Partan 2013). Considering the length of time, the perceptual systems of bees have been studied (e.g., Sprengel 1793) as well their evolutionary history and ecology, whereby behaviours have been the product of coevolving with flowering plants (Dressler 1982; Johnson and Steiner 2000; Lunau 2004), bees present a perfect opportunity as holistic models for the study of multisensory processing. Giurfa (2007) described the associative learning of honeybees as a ‘magic well’ as there are scarce examples of systems which hold the same potential in answering so many questions relating to multisensory processing. This multisensory processing raises another aspect of multimodal communication: the perceptual integration of this multisensory information through neural mechanisms, which is an active area of research (Partan 2013). There is still much to be learned about the mechanisms of multisensory integration, whereby sensory information arriving from one modality interacts and influences the processing of another modality (Talsma et al. 2010). However, it is difficult to determine the potential benefits of this multisensory integration. Detection in the presence of environmental noise is thought to be a benefit of multisensory integration, as bimodal signals may be processed faster than unimodal signals (Balkenius et al. 2009).

When visual and scent patterns overlap on the flower, they appear to be learned faster by bumblebees than patterns that do not match (Lawson et al. 2017a), due to either reinforcement of the pattern signal through multiple modalities, or signal interactions. With the disruption of one floral signal, recognition of a multimodal display will normally depend on the strength of learning of other undisrupted floral signals (Dyer and Chittka 2004a; Kaczorowski et al. 2012; Lawson et al. 2017b). When learning transfer occurs between overlapping patterns, some level of learning is maintained when one pattern is disrupted, reducing the impact of this signal disruption. Multimodal signals will be particularly effective if pollinators are able to transfer what they have learnt to novel situations, such as a direct transfer of learnt patterns between different sensory modalities, without learning occurring in the new modality. Lawson et al. (2018) demonstrated exactly this, where bumblebees conditioned to a cross-shaped scent pattern showed a preference to unscented flowers presenting matching visual patterns over circular visual patterns, despite having never encountered these visually patterned flowers before. If patterns in different sensory modalities are complementary (which may be the case where scent patterns match visual nectar guides), being able to switch between sensory modalities once a pattern has been learnt in one would be an efficient way of rapidly identifying new foraging sites in a noisy environment.

Given that bumblebees are able to transfer the learning of nonvisual scent patterns to spatially similar unlearnt visual patterns (Lawson et al. 2018), it is possible that they are able to make transfers from learnt patterns to unlearnt patterns in the other sensory modalities (where the learnt and unlearnt patterns are spatially similar but stimulate different sensory systems in the bee). We hypothesise here that this might be possible between visual and temperature patterns, which are likely to show complementary spatial patterns on the flower surface. Many flower species vary in floral temperature, and temperature can be patterned across the flower surface in many different ways (Harrap et al. 2017). Bumblebees can detect differences in temperature (Dyer et al. 2006; Whitney et al. 2008) and are able to learn to discriminate between artificial flowers that differ in the patterns of temperature that their surfaces present (Harrap et al. 2017). Most plants do not metabolically raise the temperature of their flowers, and floral temperature is largely dictated by the ability of the flower to intercept solar radiation (Herrera 1995; Totland 1996; Zhang et al. 2010). This gives a mechanistic link between colour patterns and temperature patterns, where dark-coloured areas of flowers reach a higher temperature than paler areas, creating temperature patterns that often correspond with colour patterns (Kay et al. 1981; Sapir et al. 2006; Rejšková et al. 2010; Dietrich and Körner 2014). This means that there is a frequent association between floral temperature patterns and dark-coloured visual patterns, giving a complementary multimodal signal. Here, we present two experiments that test whether bumblebees can transfer learnt temperature patterns to unlearnt complementary visual patterns (expanding on the results presented by Harrap et al. 2017), and whether they can transfer learnt visual patterns to unlearnt complementary temperature patterns.

## Materials and methods

### Artificial flower design

**Fig. 1:**
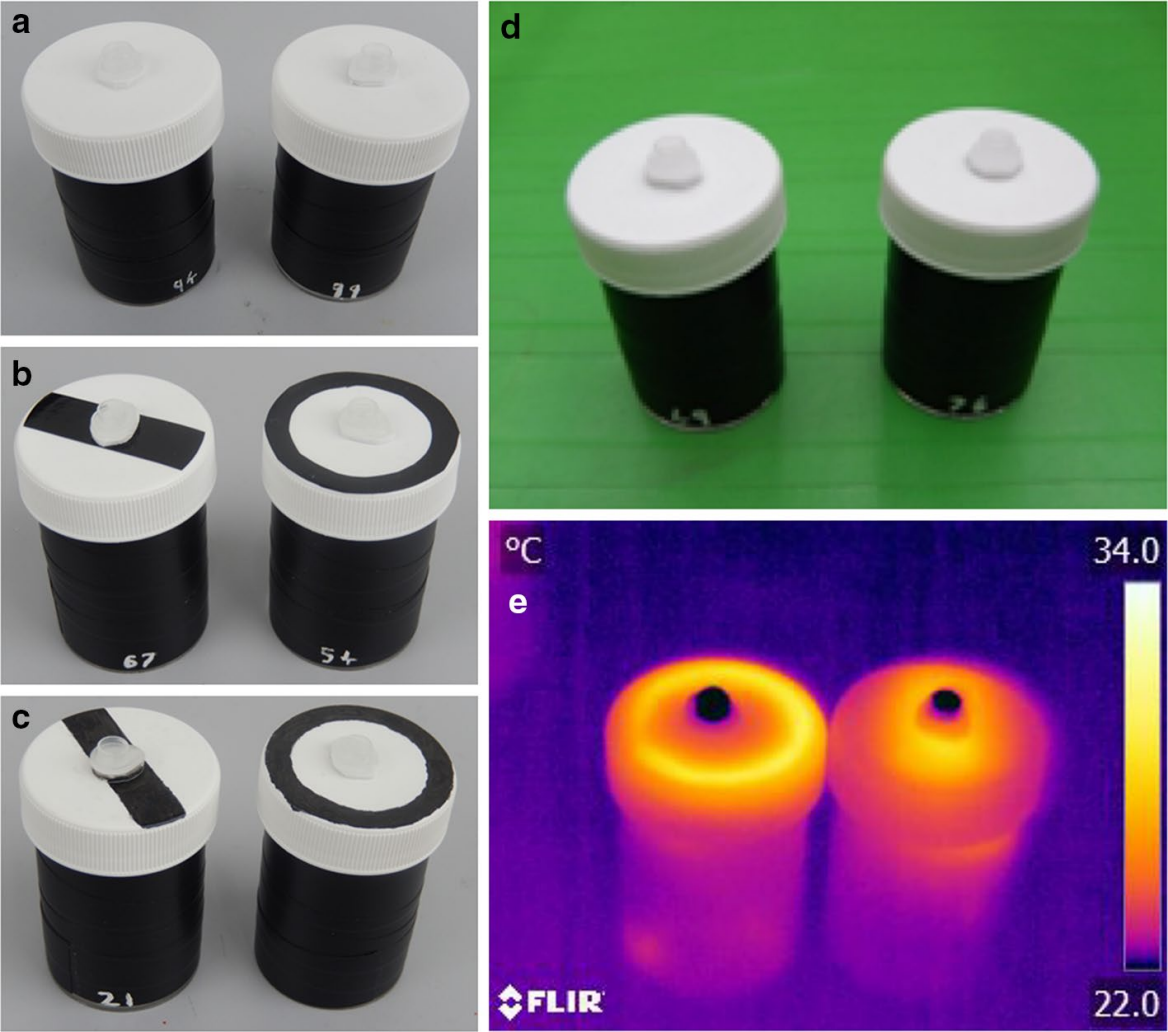
The visual pattern artificial flowers used in our experiments. **a** The control artificial flowers that present no visual pattern. **b** The visual pattern artificial flowers used in learning experiments. **c** The visual pattern artificial flowers used in both test phases. **d** The temperature pattern flowers—note that there is no visual differentiation between the two types presented. **e** Thermographs of the two flowers shown in **d**—the left flower shows a ‘circle’ pattern, and the right shows a ‘bar’ pattern. **d** Copyright and reproduced from Harrap et al. (2017) under a Creative Commons Attribution license

Three artificial flower types were used: temperature pattern flowers, visual pattern flowers, and test phase visual pattern flowers. All variants of the visual artificial flowers used in this experiment can be seen in Fig. 1a–c. Temperature pattern flowers can be seen in Fig. 1d. Control artificial flowers were identical to the artificial flowers but with the corresponding cue removed (Fig. 1a, d).

The temperature pattern which artificial flowers are used in this experiment is the ‘small artificial flowers’ described in full detail in Harrap et al. (2017). They used resistance wire arranged in a pattern underneath the flower surface, and connected in a closed circuit to a battery to generate heat. These artificial flowers create a 3 cm^2^ temperature pattern in either a ‘circle’ around the edge of the flower’s lid or a ‘bar shape’ across the flower’s centre. These artificial flowers reach an average temperature of 33 °C above the heated parts and 25 °C above the nonheated parts (Fig. 1e): This difference of about 8 °C is not unrealistic, as there are numerous patterned species of plant that show larger differences across the flower surface (Harrap et al. 2017). Removing the batteries from these artificial flowers created control flowers at the ambient temperature of the flight arena, which presented no raised temperature pattern.

Visual pattern artificial flowers were constructed from specimen jars [Thermo Scientific Sterilin, (Newport, UK), PS 60 ml, with white plastic lids] that were covered with black electrical tape and marked with randomly generated numbers for identification. An upturned 0.5 ml Eppendorf tube lid (Hamburg, Germany) with a 1 mm-thick section of heat-insulating white plastic foam stuck to its underside was glued to the centre of the artificial flower’s lid. The tube lid functioned as a feeding well.

Visual pattern flowers differed in the patterns displayed on the surface of the flower (the white lid of the specimen jar). Control flowers had no marking (Fig. 1a). Patterned flowers presented in the learning phase of the experiment had marks created by sticking a 3 cm^2^ section of black adhesive plastic (d-c-fix^®^ adhesive film, Hornschuch group, Weissbach, Germany) onto the lid. These sections of plastic were either in a circle shape about the edge of the flower’s lid or a bar shape across the flower’s centre (Fig. 1b), corresponding to the same regions heated in the temperature pattern flowers (Fig. 1e). The patterned flowers that were presented in the test phase of the experiment had their visual patterns marked onto the corresponding regions using a black permanent marker instead of cut plastic, to avoid presenting tactile stimuli and avoid any previous learning of (or innate preference for) a tactile element. Black patterns were chosen to loosely mimic the patterns seen on species of *Cistus* that showed large differences in temperature across the floral surface (Harrap et al. 2017). We note that although the ink may have presented an odour cue to the bumblebees, we attempted to apply equal quantities of ink to the patterned flowers (echoing to the carefully controlled marking done on natural plants by Hansen et al. 2012), and the patterned flowers were created days before the experiment was conducted.

### Bee experiments

Flower-naïve bumblebees, *Bombus* (*Bombus*) *terrestris* subsp. *audax* (Harris 1776), were supplied by Biobest (Westerlo, Belgium) via Agralan (Swindon, UK). Nestboxes were attached to a foraging arena via a gated tunnel, which allowed the experimenters to control which individuals were able to enter and leave the arena. Bees were individually marked with paints to aid identification. Bumblebee husbandry and foraging arenas are described in detail elsewhere (Harrap et al. 2017; Pearce et al. 2017; Lawson et al. 2018). Established differential conditioning techniques were used to explore whether bumblebees could differentiate between artificial flower types (Dyer and Chittka 2004b; Whitney et al. 2008, 2009a, 2016; Raine and Chittka 2008; Clarke et al. 2013; Lawson et al. 2018). This experiment makes use of the same bumblebees that were conditioned to the ‘small temperature patterns’ reported in Harrap et al. (2017), immediately following the nonrewarding test phase described in that paper. We also used an additional naïve set of bumblebees that were conditioned to visual patterns.

### Temperature-to-visual cross-modality test

During conditioning, the bumblebees were presented with a choice of flowers distributed randomly across the floor of the foraging arena, and allowed to forage freely and return to their nestbox at will. The numbers of bees tested in all the experiments are given in Table 1. Each bumblebee was presented with 16 flowers—eight of the rewarding temperature pattern described in Table 1 (sets 1 and 2) and eight of the corresponding nonrewarding pattern. The feeding wells of the rewarding flowers were filled with 25 µl of 30% (volume to volume) sucrose solution, and those of the nonrewarding flowers contained 25 µl of water. In the corresponding control (set 3 in Table 1), the eight rewarding and eight nonrewarding flowers were identical in appearance and did not have a temperature pattern. Flowers were not disturbed, whilst the bee was in the foraging arena, but were emptied and refilled when it was in its nestbox. Flowers were swabbed with ethanol to remove any scent marks (Stout and Goulson 2001; Pearce et al. 2017). Temperature patterns were also monitored regularly with a thermographic camera (Harrap et al. 2018), and any flowers showing faults in pattern presentation were replaced.

**Table 1:** The sequence of the experiments for all test groups in both sets of bumblebees and the flowers presented to them

Bee set	Test group	Number tested	Learning phase (60 visits)		Nonrewarding test (20 visits)		Retraining (20 visits)		Cross-modality test phase (20 visits)	
Temperature to visual	Circle rewards	12	Rewarding*	Circle T	Nonrewarding*	Circle T	Rewarding	Circle T	Nonrewarding	Circle testV
			Nonrewarding*	Bar T	Nonrewarding*	Bar T	Nonrewarding	Bar T	Nonrewarding	Bar testV
Temperature to visual	Bar rewards	12	Rewarding*	Bar T	Nonrewarding*	Bar T	Rewarding	Bar T	Nonrewarding	Bar testV
			Nonrewarding*	Circle T	Nonrewarding*	Circle T	Nonrewarding	Circle T	Nonrewarding	Circle testV
Temperature to visual	Control	12	Rewarding*	No T	Nonrewarding*	No T	Rewarding	No T	Nonrewarding	Circle testV
			Nonrewarding*	No T	Nonrewarding*	No T	Nonrewarding	No T	Nonrewarding	Bar testV
Visual to temperature	Circle rewards	12	Rewarding	Circle V	Nonrewarding	Circle testV	Rewarding	Circle V	Nonrewarding	Circle T
			Nonrewarding	Bar V	Nonrewarding	Bar testV	Nonrewarding	Bar V	Nonrewarding	Bar T
Visual to temperature	Bar rewards	12	Rewarding	Bar V	Nonrewarding	Bar testV	Rewarding	Bar V	Nonrewarding	Bar T
			Nonrewarding	Circle V	Nonrewarding	Circle testV	Nonrewarding	Circle V	Nonrewarding	Circle T
Visual to temperature	Control	12	Rewarding	No V	Nonrewarding	No V	Rewarding	No V	Nonrewarding	Circle T
			Nonrewarding	No V	Nonrewarding	No V	Nonrewarding	No V	Nonrewarding	Bar T

Sections marked with an asterisk ‘*’ are described and recorded in Harrap et al. (2017). Artificial flower types are listed as follows: ‘circle T’, circle temperature pattern; ‘bar T’, bar temperature pattern; ‘no T’, control temperature pattern; ‘circle V’, circle learning visual pattern; ‘bar V’, Bar learning visual pattern; ‘no V’, control visual pattern; ‘circle testV’, circle test visual pattern; ‘bar testV’, bar test visual pattern

As detailed in Harrap et al. (2017), we considered a bee to have ‘landed’ on a flower if it made physical contact (even if it did not quit flying). Bumblebees were observed for 60 flower landings, and we recorded whether the bee drank from the feeder or not. A landing was recorded as ‘correct’ if the bee landed on a rewarding flower and extended its proboscis into the well (to drink or probe), or if it landed on a nonrewarding flower and did not extend its proboscis (see also Whitney et al. 2008, 2009a; Clarke et al. 2013; Lawson et al. 2018). A landing was recorded as ‘incorrect’ otherwise. If the bee had depleted the feeding well, subsequent visits to the flower were discounted until the well could be replenished (once the bee had returned to the nestbox). Learning success is described and analysed in Harrap et al. (2017). It shows that bumblebees were able to learn to differentiate between temperature patterns, but were unable to differentiate between differently rewarded control flowers.

Following the initial learning phase, bees were tested to determine whether they had learnt to differentiate between the rewarding and nonrewarding stimuli using a ‘nonrewarding test’. As in the learning phase, a bee was presented with eight flowers of the ‘rewarding’ pattern and eight flowers of the ‘unrewarding’ pattern that it had been trained to, but both patterns only contained 25 µl water in their well, meaning that there was no reward to the bee. The behaviour of the bee was recorded over 20 consecutive landings. The results of this test phase are described in Harrap et al. (2017) and demonstrate that the bees continued visiting the flowers that presented the learnt rewarding stimulus, whilst remaining unable to detect any difference in the control flowers.

Having completed this nonrewarding test, the bees entered a ‘retraining phase’, where they were presented with identical stimuli to their initial learning phase (Table 1, sets 1–3) to refresh any associations made between flower signals and rewards that may have been lost during the nonrewarding test. Bees were allowed to forage freely, returning to the hive as required, until they completed at least 20 flower visits and returned to the hive.

After completing retraining, the bee was allowed to begin the ‘cross-modality learning test’. Bees (including those in the control group) were presented with 16 test visual pattern flowers (eight of each visual pattern) in the flight arena. These artificial flowers were nonrewarding with 25 µl of water, regardless of the visual pattern which they presented. Bees were allowed to forage freely until they completed 20 flower visits.

Behaviour at the flower at each landing was recorded in a similar manner to the earlier phases. During the cross-modality learning test, bee landings were classed on their response to the circle pattern. A ‘positive response’ was classed as either landing on a circle pattern flower and probing the feeder, or landing on the bar pattern flower and leaving before feeding on the feeder. Landing on a circle pattern flower and leaving without probing the feeding well or landing on a bar pattern flower and probing the feeding well was classed as a ‘negative response’. For each bumblebee, the circle pattern response rate, the proportion of the 20 flower visits that showed a positive response to the circle pattern, was calculated. When compared to the control group, if bees showed a higher circle response rate in the circle rewards pattern test group or a reduced circle response rate in the bar rewards test group, this would show a cross-modality learning between visual and temperature patterns.

Circle pattern response rate was bound between 0 and 1, and was arcsine square-root transformed for all the analyses. The circle pattern response rate in the cross-modality learning phase was compared across different test groups using the analysis of variance (ANOVA). In addition, the correlation between each bee’s success rate in the temperature pattern nonrewarding test and the circle pattern response rate in the cross-modality test phase was also compared using the analysis of covariance (ANCOVA), including the test group as a categorial variable.

### Visual-to-temperature cross-modality test

Flower-naïve bumblebees underwent an identical learning phase to that described above, except that the bees used here were presented with the learning-phase visual pattern artificial flowers, instead of temperature pattern flowers. Individual bees were assigned test groups, as shown in sets 4–6 of Table 1. No bee used in the temperature-to-visual pattern experiment was also used in the visual-to-temperature pattern experiment. Numbers of experimental animals used in all the experimental groups are given in Table 1.

The learning phase of experiments and recording of bee landing and probing behaviours were carried out as described previously. Bees then undertook a nonrewarding test phase, where they were presented with nonrewarding test phase visual artificial flowers or control flowers, dependent on test groups. As in the temperature pattern experiment, bees were allowed to forage freely for 20 flower visits, and landing and probing behaviour was observed and recorded. Because the test phase flowers had their marks produced with ink, as opposed to plastic, this meant that test artificial flowers could not be washed with ethanol between foraging bouts. Test flowers were instead wiped with a dry cloth and set aside for at least a few hours between bees’ test phases (during learning phases). Although bee scent marks may remain on the polypropylene lids for a period of time, we suggest that the time which they were left for should have avoided lingering scent providing confounding cues in the experiment, given that scent marks have a lifespan of around 40 min on natural floral surfaces (Stout and Goulson 2001).

Following completion of the test phase, bees were presented with rewarding and nonrewarding learning-phase visual artificial flowers or control flowers as determined by test group (Table 1 sets 4–6), and allowed to forage in a retraining phase. Following at least 20 flower visits in the retraining phase, bees were allowed to begin the cross-modality learning test phase upon beginning the nest foraging bout. This test phase was carried out as described above except that bees were presented with nonrewarding temperature pattern flowers, instead of nonrewarding visual pattern artificial flowers. Visitation and feeding well-probing responses were again recorded during the cross-modality learning test phase.

Success rate over the previous ten visits (starting at visit 10, then 20, 30, etc.) in the learning phase and overall success rate in the nonrewarding test phase were calculated, based on whichever visual pattern had been rewarding in that test group. Circle pattern response rate was calculated for the cross-modality test phase, as described for the previous experiment. However, in this experiment, the circle pattern response rate described the responses to the visual patterns in the learning and nonrewarding test and temperature patterns in the cross-modality learning test. Success rate for both learning and nonrewarding test phases was analysed by dividing the visit data into ten-visit intervals, and comparing these with an ANOVA. If the ANOVA demonstrated a significant difference between the three treatments, post hoc paired *t* tests were conducted, assuming a Bonferroni correction. Circle pattern response rate was analysed as described above in the ‘temperature-to-visual cross-modality test’.

## Results

### Temperature-to-visual cross-modality test

**Fig. 2:**
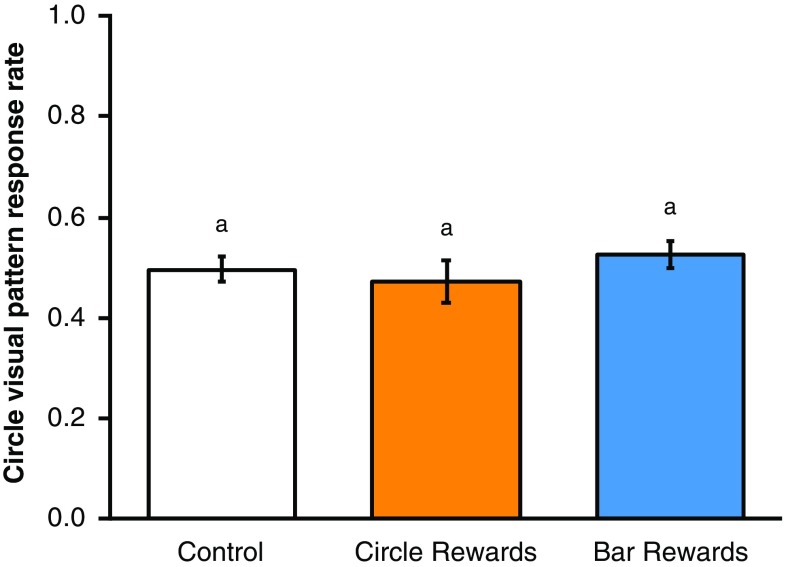
The mean circle pattern response rate ± SEM of bumblebees conditioned in our temperature pattern experiment when presented with matching visual patterns in our cross-modality learning test, ordered by test group. Letters above bars denote groups as defined by post hoc Tukey’s test where *p* < 0.05

**Fig. 3:**
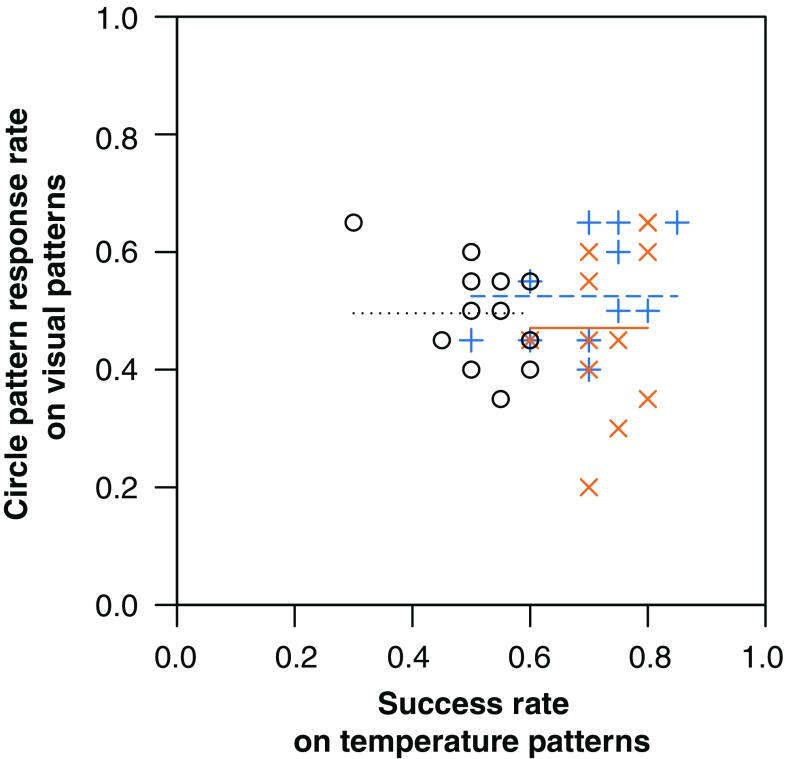
The relationship between success rate of bumblebees in a nonrewarding test of conditioning to temperature patterns and the circle pattern response rate when those bees are presented with matching visual patterns. Lines indicate the average circle pattern response rates for each test group. Shape of points and dashing of lines corresponds with what temperature pattern which the bee was conditioned on (their test group), as does the colour of both. Black circles, o, and dotted line, control group (no temperature pattern conditioning); orange crosses, x, and solid line, circle rewards (bees conditioned to circle temperature patterns); blue plus signs, +, and dashed line, bar rewards (bees conditioned to bar temperature patterns)

Bumblebees conditioned to temperature patterns did not show any cross-modality pattern learning when presented with matching visual patterns or control equivalents in the cross-modality learning test (ANOVA, *F*_2,33_ = 0.75, *p* = 0.482, Fig. 2). When presented with the visual patterns, the bees appeared to forage randomly, achieving response rates of c. 50% regardless of whether they were conditioned to bar, circle or no temperature patterns (the control group). Bees in the control group showed a mean response rate in the cross-modality learning phase of 49%, suggesting that they have no strong preference for either visual pattern. The success which a bee achieved when its conditioned stimulus (bar temperature patterns, circle temperature patterns or no patterns, dependent on test group) was tested was not correlated with the response rate shown in the visual pattern test, regardless of which test group the bee was in (ANCOVA: interaction term, *F*_2,30_ = 2.51, *p* = 0.098; test group term, *F*_2,30_ = 0.97, *p* = 0.392; success in temperature pattern test, *F*_1,30_ = 0.09, *p* = 0.769, Fig. 3). These findings suggest that temperature pattern learning does not inform recognition and learning of matching visual patterns.

### Visual-to-temperature cross-modality test

**Fig. 4:**
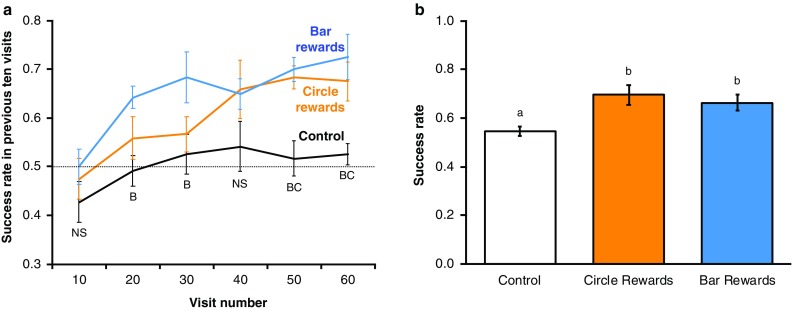
Bumblebee learning when foraging on visual pattern artificial flowers. **a** The relationship between foraging success and experience of the artificial flowers (flower visits made) during the learning phase. The dotted line indicates the 50% success level. Solid lines indicate the mean foraging success (± SEM) of bees in the previous ten visits. Colour and label of solid lines and error bars correspond with test group: black, the control group; orange, circle rewards group; blue, bar reward group. Letters below bottom error bars report post hoc pairwise *t* tests comparing the treatments in the ten-visit block (the associated ANOVA results are reported in the Supplementary Information): ‘NS’ reports no significant difference between the treatments; ‘B’ reports that the bar reward group differed significantly (*p* < 0.05) from the other two groups; ‘BC’ reports that the bar and the control reward groups differed significantly from the control group (*p* < 0.05), but did not differ significantly from each other. **b** The mean foraging success (± SEM) of bees in different test groups during the nonrewarding test phase. Letters above bars denote groups as defined by post hoc Tukey’s tests where *p* < 0.05. Twelve bumblebees completed this experiment in each test group (36 bees in total across three nests)

When foraging on visual pattern cues in the learning phase, the bumblebees learnt to distinguish flowers when presented with visual pattern cues but not in the control group (Fig. 4a; ANOVA results presented in Supplementary Information). Bees completed the learning phase in 4.96 ± 0.30 bouts (mean ± SD) making 12.93 ± 0.68 landings per bout. When conditioned visual pattern stimuli were tested, bees presented with visual patterns (the circle rewards and bar rewards) achieved a greater success rate than those in the control group (ANOVA, *F*_2,33_ = 6.18, *p* < 0.01, Fig. 4b).

**Fig. 5:**
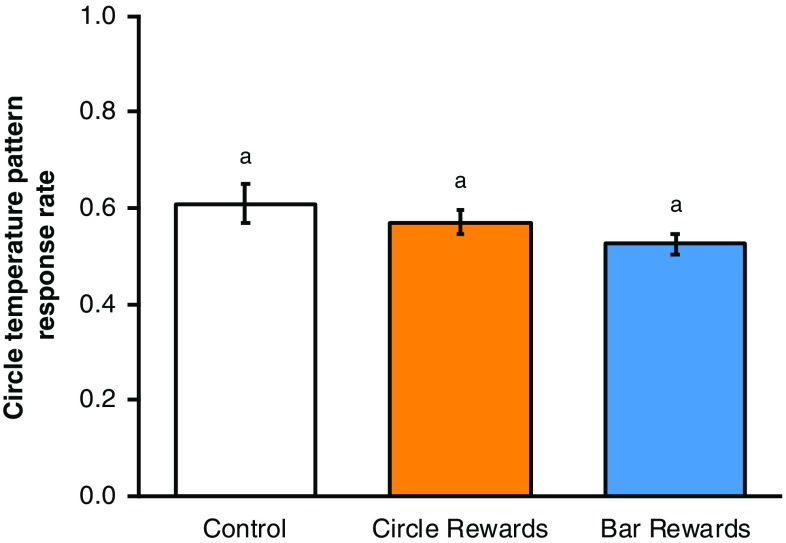
The mean circle pattern response rate (± SEM) of bumblebees conditioned in the visual pattern experiment when presented with matching temperature patterns in the cross-modality learning test, ordered by test group. Letters above bars denote groups as defined by post hoc Tukey’s test where *p* < 0.05

**Fig. 6:**
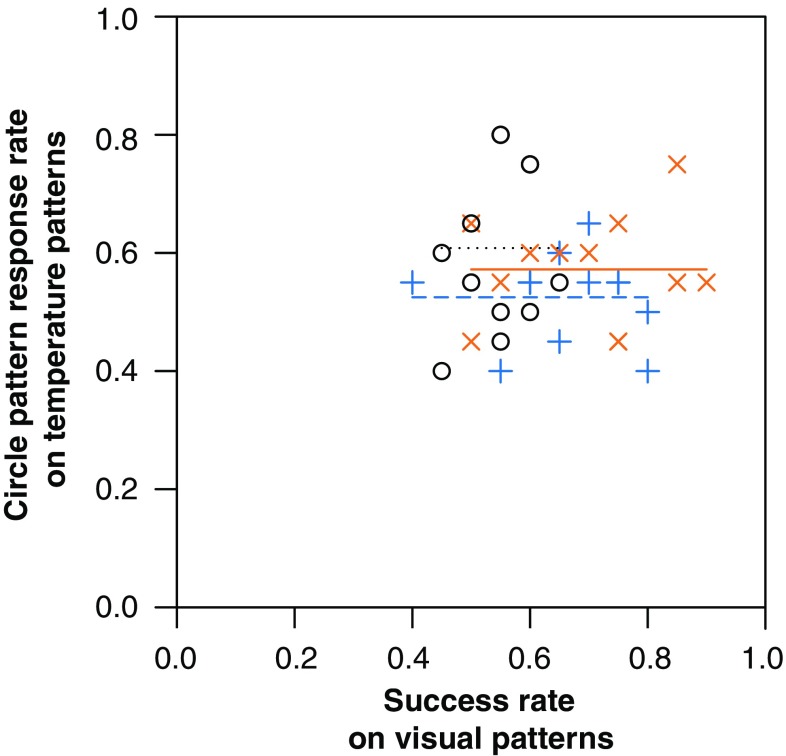
The relationship between success rate of bumblebees in a nonrewarding test of conditioning to visual patterns and the circle pattern response rate when those bees are presented with matching temperature patterns. Lines indicate the average circle pattern response rates for each test group. Shape of points and dashing of lines corresponds with what visual pattern which the bee was conditioned on (their test group), as does colour of both. Black circles, o, and dotted line, control group (no temperature pattern conditioning); orange crosses, x, and solid line, circle rewards (bees conditioned to circle temperature patterns); blue plus signs, +, and dashed line, bar rewards (bees conditioned to bar temperature patterns)

When bees conditioned to visual patterns were presented with artificial flowers with the corresponding temperature patterns, bees did not appear to show a response that was altered by their prior conditioning in the visual modality (Fig. 5). Test group identity had no effect on the circle pattern response rate (ANOVA, *F*_2,33_ = 1.961, *p* = 0.157). Bees in the control group, which had no prior conditioning to visual patterns, showed a mean circle pattern response rate of 61%, suggesting that bees had a preference for the circle temperature pattern over the bar. This preference is reflected in similar foraging choices of the other test groups (Fig. 5). The success rate bees achieved in the first test phase had no influence on the later circle response rate (ANCOVA: interaction term, *F*_2,30_ = 0.59, *p* = 0.561; test group term, *F*_2,30_ = 2.01, *p* = 0.152; success in visual pattern test, *F*_1,30_ = 0.06, *p* = 0.814, Fig. 6).

## Discussion

Our experiments suggest that cross-modal transfer of learnt patterns does not appear to occur in either direction between temperature and visual patterns. Bumblebees conditioned to temperature patterns appeared to forage randomly when presented with matching dark visual patterns (Fig. 2). Likewise, bees conditioned to visual patterns when presented with matching temperature patterns showed a consistent response to their preferred circle temperature pattern (Fig. 5). Bumblebees were capable of learning and recognising the visual and temperature patterns presented to them (Fig. 4, and the results presented in Harrap et al. 2017). Therefore, it is unlikely that the lack of cross-modality transfer of pattern learning observed here is the result of the bees’ inability to detect any of the patterns presented to them. Such transfers in pattern learning have been seen to occur with bumblebees conditioned to scent patterns when presented with matching visual patterns (Lawson et al. 2018). Our current results suggest that such cross-modality learning transfer is not universal across all patterned signal modalities and seems to only occur between specific patterned signals.

The nonlearnt sensory pattern transfer described by Lawson et al. (2018) may be explained by the morphology of the bumblebee’s brain. We assume that bumblebee thermoreception primarily involves sensilla on the antennae (Fialho et al. 2014), but it could also be occurring on the other areas of the body, as is known in the other insects (Abram et al. 2017). Scent and sight have close neurological links (Leonard and Masek 2014). Neural pathways from hymenopteran antennal and optic lobes, which carry scent and sight signals, respectively, meet at the mushroom body calyx (Gronenberg 1999, 2001), where some structures receive information from both pathways (Mobbs 1982; Gronenberg 2001; Ehmer and Gronenberg 2002). Consequently, the spatial arrangement of visual patterns may elicit similar stimulation as a matching scent pattern in the bee’s brain. Mushroom bodies are strongly associated with memory formation and learning (Menzel 2001; Davis 2005). Bees are, therefore, likely to process scent and sight memories together (Leonard and Masek 2014), which might allow the cross-modality learning observed (Lawson et al. 2018).

Alternatively, our failure to achieve pattern learning between temperature and visual patterns may be a consequence of how the bumblebees are physically interacting with temperature patterns. When a bumblebee lands on a flower with a temperature pattern alone, it may not learn the pattern as a whole. It may, for example, learn to associate the hot edges of the display with rewards, without ‘visualising’ the entire circular ring pattern of temperature across the whole flower. Thus, the corresponding visual pattern is not recognised as matching the conditioned temperature pattern and vice versa. However, bees did not require such patrolling of the flower surface to associate a scent pattern with a matching visual pattern and still achieved cue transfer (Lawson et al. 2018). In addition, Harrap et al. (2017) have previously demonstrated that bumblebees were able to distinguish temperature patterns of different shapes in a similar location. Floral orientation could also be important for both the presentation of patterns of different sensory modalities and the ability of the pollinator to interact with the surface (Whitney et al. 2009a; Rands et al. 2011), and it may be that presenting our patterns horizontally rather than vertically would allow the bees to distinguish the heat patterns with a different degree of ease and accuracy, altering these patterns’ use as a multimodal signal.

The lack of any apparent cross-modality pattern-learning responses between visual and temperature patterns does not exclude the possibility that bumblebees show such pattern-learning transfer with the other overlapping patterns or with different modalities. Floral temperature patterns often correspond with other signal modalities, and can be influenced by the aspects of the flower surface structure. These may be due to texture influencing both heat loss by trapping air (Miller 1986) and the flower’s surface area, which in turn affects water loss and light inception (Whitney et al. 2011). Texture signals are detected across the tarsi and antennae of bees (Kevan and Lane 1985; Whitney et al. 2009a), as is temperature (Heran 1952; Whitney et al. 2008). Texture and temperature patterns might, therefore, induce more similar stimulations to the bee than the temperature and visual patterns that we describe here. Texture-related signals often overlap with visual signals, and this is particularly true where visual patterns are the result of structural aspects of the petal surface, such as floral iridescence (Whitney et al. 2009b, c; Kjernsmo et al. 2018) or gloss (Glover and Whitney 2010; Whitney et al. 2012).

In this study, we investigated the capacity of bumblebees experiencing matching temperature and visual patterns to show cross-modality pattern-learning transfer similar to those described by Lawson et al. (2018) between scent and visual patterns. We show that similar pattern-learning transfer is not universal among floral modalities. The lack of learning transfer between temperature and visual patterns appears to be due either to how bees detect and interact with the temperature patterns, or to differences between the neurological pathways by which temperature, vision, and scent learning takes place in bees. It may also be that some of these modalities such as thermal patterns work more effectively as a back-up cue when primary cues such as visual patterns are uninformative, following the efficacy back-up hypothesis (Hebets and Papaj 2005; Leonard et al. 2012; Lawson et al. 2017b). Despite not finding such cross-modality learning, we have gained important information about how different floral signals might be experienced by bees visiting multimodal displays and how transfer of pattern learning occurs.

## Electronic supplementary material

Below is the link to the electronic supplementary material.

Supplementary material 1 (ZIP 88 KB)
