# Supplementary material for: Cross-modal transfer in visual and nonvisual cues in bumblebees
Source: J Comp Physiol A Neuroethol Sens Neural Behav Physiol. 2019 Mar 11;205(3):427–37. doi: 10.1007/s00359-019-01320-w (PMC6579774; doi:10.1007/s00359-019-01320-w)
Supplement: Supplementary file 1 — Supplementary material 1 (ZIP 88 KB) [file 359_2019_1320_MOESM1_ESM.zip › Users/frsar/Documents/Harrap_et_al_supplementary/supplementary_results_figure_4a.pdf]

Supplementary Information. Harrap, Lawson, Whitney & Rands "Cross-modal transfer in visual and non-visual cues in bumblebees" *Journal of Comparative Physiology A*

			p from *post hoc* paired *t*-tests		
**Visit number**	$F_{2,33}$	p	**bar *v* circle**	**bar *v* control**	**circle *v* control**
10	0.71	0.497	—	—	—
20	5.00	0.013	0.267	**0.010**	0.510
30	3.58	0.039	0.198	**0.044**	1.000
40	1.77	0.186	—	—	—
50	12.21	< 0.001	1.000	**< 0.001**	**< 0.001**
60	7.84	0.002	1.000	**0.002**	**0.022**

Figure 4a supplementary information. Statistical summary of the ANOVA tests conducted to explore differences in learning speed during the visual-to-temperature learning phase.
