# Supplementary material for: Cross-modal transfer in visual and nonvisual cues in bumblebees
Source: J Comp Physiol A Neuroethol Sens Neural Behav Physiol. 2019 Mar 11;205(3):427–37. doi: 10.1007/s00359-019-01320-w (PMC6579774; doi:10.1007/s00359-019-01320-w)
Supplement: Supplementary file 1 — Supplementary material 1 (ZIP 88 KB) [file 359_2019_1320_MOESM1_ESM.zip › Users/frsar/Documents/Harrap_et_al_supplementary/readme.pdf]

Key to Source data

Data are organised by bee sets 1, 2 and 3 (the temperature to visual experiment) and bee sets 4, 5 and 6 (the Visual to temperature experiments), as described in the main text (table 1).

Raw data

Provides the landing action data for each visit of each of the bees that completed the experiment. Provided for every experiment phase for bee sets 4, 5 and 6 and the non-rewarding test phase of bee set 1, 2, and 3. For data from the learning and non-rewarding test phases of bee sets 1, 2 and 3, see data for Harrap et al. (2017) [https://datadryad.org/resource/doi:10.5061/dryad.qp244].

Column designations are as follows:

bee: bee identifier number within the 'Temperature to Visual' and 'Visual to temperature' experiments.

colour: colourings of the bee's identity marker

date: date of testing

set: the bee set number as described in the main text. Not bee set dictates the modality onto which bees are conditioned and tested as described in the main text (table 1).

nest: The nest the bee was from. Nests are identified by the lettering indicating the workstation within the Lab their flight area was on. If a nest is replaced a number is added to the workstation letter (*e.g.* D2).

test group: The test group the bee was in, as described in the main text. Note that the test group a bee is in dictates which cues correspond with the rewarding and nonrewarding in the test and learning phases and those bees are presented in the test phases.

Columns beyond the column labelled 'visit' list the foraging actions of each bee. Numbers of columns beyond this point correspond to the flower visit number. Letters correspond to bumblebee foraging actions as follows. In learning and test phases: 'S' landing on a rewarding flower but no probing. 'S+' landing on a rewarding flower and feeding well is probed. 'W' landing on a nonrewarding flower but no probing. 'W+' landing on a nonrewarding flower and feeding well is probed. In cross-modality test phases: 'C' landing on a circle pattern flower but no probing. 'C+' landing on a circle pattern flower and feeding well is probed. 'B' landing on a bar pattern flower but no probing. 'B+' landing on a bar pattern flower and feeding well is probed.

Learning rate data

Provides the success rate data, for the previous 10 visits for learning phase. Provided for bee sets 4, 5 and 6. For data for bee sets 1, 2 and 3, see data for Harrap et al. (2017) [https://datadryad.org/resource/doi:10.5061/dryad.qp244].

Columns 'bee', 'colour', 'date', 'nest', 'set' and 'test group' as described above. Further columns are as follows:

visit number: the visit number where success rate was calculated.

Correct actions: the number of correct foraging actions (as defined in the main text) the bee has made in the previous 10 visits

Success Rate: the success rate achieved by the bee in the previous 10 visits

Test phase data

Provides data on the success rates and circle pattern response rates for bees in the nonrewarding test phases and cross-modality test phase. Provided for all bee sets.

Columns 'bee', 'colour', 'date', 'nest', 'set' and 'test group' as described above. Further columns are as follows:

Correct actions in non-rewarding test: number of correct actions that occurred in the non-rewarding test phase (as described in main text).

Success rate in non-rewarding test: the success rate in the non-rewarding test phase (as described in main text).

Positive Circle actions in Cross-modality test phase: number of positive responses to the circle pattern that occurred in the cross-modality test phase (as described in main text).

Circle response rate in Cross-modality test phase: the circle response rate in cross-modality test phase (as described in main text).

Plotted figure values

Data plotted in figures is provided as relevant to the figures in 'Plotted data.xlsx'

Provides detail on the points plotted in respective figures. The mean and standard error of the mean of bee success rate is given for each 10 visit division for figures 4a and for the whole of the respective test phases for figures 2, 4b and 5. As stated in the document values plotted for figures 3 and 6 can be found in Test phase data for bee sets 1,2,3 and 4,5,6 respectively.
